# Prevalence and Level of Antibodies Anti-*Plasmodium* spp. in Travellers with Clinical History of Imported Malaria

**DOI:** 10.1155/2013/247273

**Published:** 2013-04-04

**Authors:** Rita Medina Costa, Karina Pires de Sousa, Jorge Atouguia, Luis Távora Tavira, Marcelo Sousa Silva

**Affiliations:** Unidade de Ensino e Investigação de Clínica Tropical, Centre for Malaria and Tropical Diseases, Instituto de Higiene e Medicina Tropical, Universidade Nova de Lisboa, Rua da Junqueira 100, 1349-008 Lisbon, Portugal

## Abstract

In this study, we show that 40.29% of travellers with a possible history of malaria exposure were positive for anti-*Plasmodium* spp. antibodies, while these individuals were negative by microscopy. The antibody test described here is useful to elucidate malaria exposure in microscopy-negative travellers from endemic countries.

## 1. Introduction

Malaria is an infectious disease caused by a protozoan parasite of the genus *Plasmodium*, which is transmitted between humans by the bite of infected female *Anopheles* mosquitoes. These parasites have a complex life cycle, both in the invertebrate vector and vertebrate hosts. In the human body, parasites multiply in hepatocytes, and then invade red blood cells (RBCs), initiating blood stage infection, which corresponds to the symptomatic period of the disease [1].

Malaria remains one of the most serious public health problems not only in endemic countries, where 2 billion people (approximately 40% of the world's population) are at risk of contracting the disease, but also in nonendemic areas, where the increasing number of imported malaria cases is worrying [2]. In developed countries, imported malaria predominates in tourists and immigrants who travel to their home countries to visit friends and relatives. Every year, approximately 125 million international travellers visit malaria endemic areas, and 30,000 of them contract the disease [3, 4]. In Portugal, the occurrence of 50 such cases per year [5] is estimated according to the National Public Health System.

Following infection with any of the five species of *Plasmodium* that are capable of infecting humans, *P. falciparum*, *P. ovale*, *P. vivax*, *P. malariae,* and *P. knowlesi*, specific antibodies are produced one or two weeks after the initial infection and persist for three to six months after parasite clearance [6]. These antibodies may endure for months or years in semi-immune patients in endemic countries where reinfection is frequent. However, in a naïve patient, antibody levels fall more rapidly. Reinfection or relapse leads to a secondary response with a high and rapid rise in antibody titres [6, 7].

Thus, in the present study, we aim to evaluate the prevalence and the level of anti-*Plasmodium* spp. antibodies in serum samples from travellers with possible clinical signals and symptoms of malaria. Using an ELISA-based commercial immunoassay kit to measure antimalarial antibodies, we determined the raw serological profile of these individuals. Additionally, we compare the latter serological profile with the gold-standard laboratory diagnosis, based on direct microscopy.

## 2. Materials and Methods

### 2.1. Study Population

The population for this study consisted of 335 individuals with possible clinical history of malaria and 23 healthy individuals (healthy Portuguese individuals who have never been in malaria-endemic countries). All of the 435 subjects who have had potential exposure to *Plasmodium* spp. travelled back to Portugal from malaria-endemic regions of Africa, Brazil, Ecuador, India, Indonesia, Thailand, and Haiti, either as residents or tourists, and most of them are adults. Subjects for this study were actively recruited after being seen for symptoms of malaria at the Clinical Unit for Tropical Diseases (IHMT, Portugal).

Following microscopic examination of Giemsa-stained blood films, subjects who were potentially exposed to the parasite and had concomitant positive microscopy were categorized into group 1 (*n* = 45); subjects potentially exposed to the parasite but displayed negative microscopy were categorized into group 2 (*n* = 290); and finally, healthy naïve subjects were categorized into group 3 (*n* = 23).

### 2.2. Microscopic Diagnosis of Malaria

From each patient was obtained blood by venipuncture (5 mL of blood in anticoagulant), and two blood smears were prepared (thick and thin blood films). The haematological data was obtained from an automatic Coulter Sysmex K-1000 analyzer (Emílio de Azevedo Campos). Both blood films were stained by Giemsa's staining method and were observed on an optical microscope. The thick blood film was used to attain a qualitative diagnosis for malarial infection, and the thin blood film was used to identify the *Plasmodium* species, when infection was present. Moreover, when infection was established, the thin blood film was also used to count the number of parasites in 200 leucocytes, and this number was then converted to number of parasites in one microliter of blood [8]. Samples with no visible parasites after scoring 100 fields were considered to be negative for this test. These procedures were used as the diagnostic test for malaria. This clinical study protocol was approved by the Institutional Ethics Committee of the Instituto de Higiene e Medicina Tropical, Universidade Nova de Lisboa, Portugal (clinical study registration 4, 2012, PN, February 2012).

### 2.3. Serological Measurement of Antimalarial Antibodies

Total anti-*Plasmodium* spp. (antimalarial) antibodies were analysed from serum samples collected from all individuals (*n* = 358). The Newmarket Laboratories Malaria EIA kit (Bio-Rad, USA) was used in this study for evaluating the prevalence of total antimalarial antibodies in the depicted groups of subjects. This system is based on the binding of anti-*Plasmodium* spp. antibodies (IgG, IgM, and IgA) by use of four recombinant antigens that detect antigens from *P. falciparum*, *P. ovale*, *P. vivax,* and *P. malariae*. The test was performed as recommended by the manufacturer, as follows. 50 *μ*L of individual undiluted sera samples were added in each single well. For each assay plate, 50 *μ*L of positive and negative controls were also dispensed. The negative control was tested in triplicate and the positive control in duplicates. After mixing on a plate shaker for 30 seconds, the plate was covered and incubated for 30 minutes at 37°C before being washed 5 times with wash buffer. 50 *μ*L of diluted horseradish peroxidase-conjugated antibody were added to each well, and the plates were incubated for 30 minutes at 37°C. The wells were washed again 5 times, and 50 *μ*L of substrate solution were added to each well. The plate was then covered and incubated in the dark for 30 minutes. Finally, 50 *μ*L of 0.5 M sulphuric acid were added to each well to stop the reaction, and the absorbance was read at 450 nm, with a reference wavelength of 620 nm. The antibody index was obtained by dividing the OD value of each sample (at 490 nm) by the cut-off value, which was calculated as the mean of the negative control value plus 0.100, according to the manufacturer.

### 2.4. Data Analysis

After establishing the study groups, a commercial immunoassay Malaria EIA kit (Bio-Rad, USA) was used to determine total anti-*Plasmodium* spp. antibodies. The Malaria EIA kit is based on presence of antibodies (IgM, IgG, and IgA) reactive to four recombinant antigens to detect *P. falciparum*, *P. ovale*, *P. vivax,* and *P. malariae*. The cut-off value was calculated as the mean of the negative control value plus 0.100. To validate the assay, the optical density (OD) of each negative control should be lower or equal to 0.080, and the OD of each positive control should be greater than or equal to 1.000. The antibody index was obtained by dividing the OD value of each sample (at 490 nm) by the cut-off value. The samples with indexes lesser than or equal to 1 were considered negative. Moreover, the samples with an antibody index greater than 1 were considered positive for the presence of antimalarial antibodies. This immunoassay does not distinguish between IgG, IgM, and IgA antibodies, or between antibodies to *P. falciparum*, *P. vivax*, *P. ovale,* and *P. malariae*.

## 3. Results and Discussion

In this study, the prevalence and level of total antimalarial antibodies (anti-*Plasmodium* spp.) were determined in patients with possible clinical history of malaria. These patients were actively recruited in the Clinical Unit for Tropical Diseases (IHMT, Portugal). The malaria antibody EIA (Newmarket, UK; Bio-Rad) is based on binding of anti-*Plasmodium* antibodies present in a serum sample to antigens immobilized on a solid phase. The antigens are four recombinant types specific for *P. falciparum* with cross-reactivity for *P. ovale* and *P. malariae* and one specific antigen for *P. vivax*. The test detects total immunoglobulin (Ig) antibodies against *P. falciparum* and *P. vivax* and shows 80% cross-reactivity with *P. ovale* and 67% with *P. malariae*. The malaria antibody EIA was performed as recommended by the manufacturer.

Table 1 shows the distribution and characterization of two groups of individuals potentially exposed to *Plasmodium* spp. based on microscopic diagnosis for malaria (blood films), compared with a third group consisting of nonexposed individuals.

**Figure 1:**
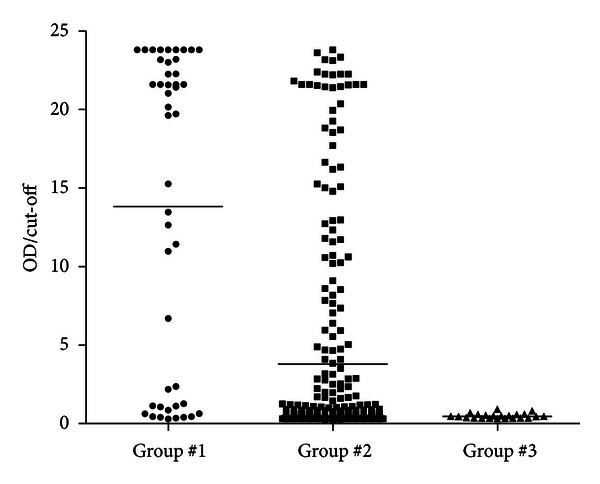
Distribution of antimalarial antibodies in subjects with possible clinical history of imported malaria. Antibody index represents the ratio OD/cut-off for each sample. Group no. 1: travellers potentially exposed to *Plasmodium* spp. and microscopically positive for malaria (*n* = 45); group no. 2: travellers potentially exposed to *Plasmodium* spp. and microscopically negative for malaria (*n* = 290); and group no. 3: control, healthy subjects (*n* = 23).

Figure 1 and Table 1 show the distribution and level of total antimalarial antibodies in three groups: group no. 1 (travellers potentially exposed to *Plasmodium* spp. and microscopically positive for malaria), group no. 2 (travellers potentially exposed to *Plasmodium* spp. and microscopically negative for malaria), and group no. 3 (nonexposed individuals, healthy individuals who reside in Portugal). From all individuals potentially exposed to *Plasmodium* spp. (*n* = 335), 13.43% (*n* = 45) had positive blood films. On the other hand, 40.29% (*n* = 135) had positive ELISA serological reactions to *Plasmodium* spp. (Table 1). As expected, naïve individuals (group no. 3) had serological and microscopically negative results to malaria. 

**Figure 2:**
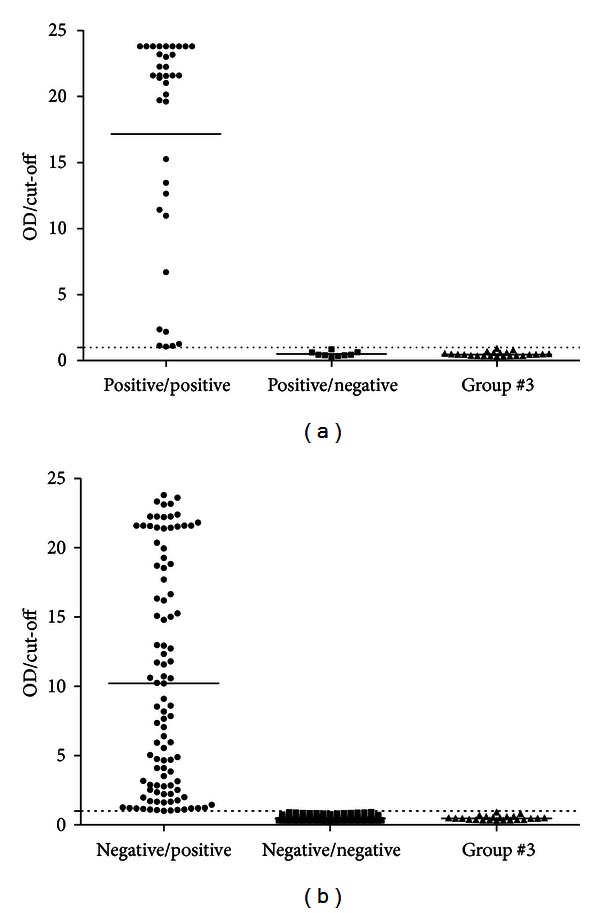
Distribution of antimalarial antibodies in subjects with possible clinical history of imported malaria. (a) (Group no. 1): individuals who were potentially exposed to the parasites and have as a positive blood film diagnostic for malaria (*n* = 45). (b) (Group no. 2): subjects potentially exposed to the parasite but have negative blood film diagnostic for malaria (*n* = 290). Group no. 3: control, healthy subjects (*n* = 23).

Results regarding the prevalence and level of antimalarial antibodies displayed by subjects in group no. 1 (positive blood film) and group no. 2 (negative blood film) are detailed in Figures 2(a) and 2(b), respectively. Both results are compared with the ones from group no. 3 (nonexposed individuals). Of all microscopically positive individuals (*n* = 45), 80% (*n* = 36) showed some level of antimalarial antibodies (Figure 2(a)). Furthermore, of all individuals microscopically negative for malaria (*n* = 290), 34.1% (*n* = 127) had antimalarial antibodies and 65.9% (*n* = 191) had no serological reaction to *Plasmodium* spp. (Figure 2(b)).

The use of immunofluorescence to determine the prevalence of antimalarial antibodies has been used to estimate malaria endemicity [9, 10], but its use was limited by dependence on cultured parasites, expensive fluorescence microscopes, and the subjective nature of slide reading. However, determination of antimalarial antibodies by ELISA has been shown to be a potentially useful epidemiological tool [11, 12]. For example, antibodies to the circumsporozoite antigen have been associated with transmission intensity [10] and with cumulative (age related) exposure to infection in both Brazil [13] and Sri Lanka [14]. In addition, serological data are relatively simple to collect: blood processed or blood spots collected. Processed blood or blood spots onto filter paper are a suitable source of serum if appropriately collected and stored [15, 16], and ELISA-based antibody assays are robust, relatively low tech with high throughput and inexpensive.

However, antibody detection is not a substitute for blood film examination in the diagnosis of an acute attack of malaria, and it is mainly used in the screening of prospective blood donors to avoid transfusion-transmitted malaria [17, 18]. A blood film should be taken from anyone whose symptoms and history of travel suggest malaria. In clinical practice, serology has no place in diagnosing acute malaria, but in certain circumstances, it can be useful for better understanding a possible history of malaria when no parasites have been found through microscopy, which is the gold standard method for the diagnosis of malaria. That may happen when a blood film from the subject was never analysed or because they had taken antimalarial drugs at a dosage sufficient to depress parasitemia to undetectable levels before the contact with the malaria parasite. In such cases, serological investigations may confirm or exclude malaria, according to previous reports suggesting that an antibody test is effective in detecting malaria infection in travellers returning from overseas [19] and in nonimmune visitors to endemic areas after their departure [19, 20].

Thirty-six out of 45 (80%) subjects from group 1 displayed levels of anti-*Plasmodium* spp. antibodies when titrated by a commercial immunoassay Malaria EIA kit, while the remaining 9 (20%) subjects had no significant levels of anti-*Plasmodium* spp. antibodies, when compared to the control group (group no. 3). These 9 individuals might be at the initial phase of infection or at an acute phase of the disease, which would explain why no antibodies have been detected in their serum, while they had microscopically positive slides or a false positive microscopically. In any of these cases, antibody levels are too low to be detected by this method or have not been yet produced. On the other hand, 200 out of 335 (66%) subjects did not show significant levels of anti-*Plasmodium* spp. antibodies with the commercial kit used in this study. Moreover, 135 (40.29%) subjects had significant levels of anti-*Plasmodium* spp. antibodies, when compared to the control group (group no. 3). In our study, the presence of antibodies in 34.1% of the subjects with negative microscopy confirms the microscopy limitations, and that an additional antibody test is an asset to know about contact with malaria parasite in travellers from endemic countries. Furthermore, it is often difficult to distinguish between species, especially if the patient has already been treated or has done chemoprophylaxis.

This work has been complemented with new studies that evaluated the serological reactivity in these patients and were identified major antigenic proteins that are responsible for the production of the antibodies detected in this study [21].

## 4. Conclusions

This study showed that 40.29% of travellers with a possible history of malaria exposure were positive for anti-*Plasmodium* spp. antibodies, while 40.29% of these individuals were negative by microscopy. The antibody test described here is useful to elucidate malaria exposure in microscopy-negative travellers from endemic countries.

## Figures and Tables

**Table 1:** Characterization of the individual groups used for the evaluation of antimalarial antibodies.

	Number of individuals (*n*)	Microscopic diagnosis	Serological diagnosis
Group no. 1	45	Positive	Positive (*n* = 36) Negative (*n* = 9)
Group no. 2	290	Negative	Positive (*n* = 99) Negative (*n* = 191)
Group no. 3	23	Negative	Positive (*n* = 0) Negative (*n* = 23)
